# Functionalizing Fibrin Hydrogels with Thermally Responsive Oligonucleotide Tethers for On-Demand Delivery

**DOI:** 10.3390/bioengineering9010025

**Published:** 2022-01-10

**Authors:** Chase S. Linsley, Kevin Sung, Cameron White, Cara A. Abecunas, Bill J. Tawil, Benjamin M. Wu

**Affiliations:** 1 Department of Bioengineering, Samueli School of Engineering, University of California, Los Angeles, CA 90095, USA; niveksung@gmail.com (K.S.); cameron.white49@gmail.com (C.W.); abecunas@umich.edu (C.A.A.); btawil@seas.ucla.edu (B.J.T.); 2 Division of Advanced Prosthodontics, School of Dentistry, University of California, Los Angeles, CA 90095, USA; 3 Weintraub Center for Reconstructive Biotechnology, School of Dentistry, University of California, Los Angeles, CA 90095, USA; 4 Department of Materials Science & Engineering, Samueli School of Engineering, University of California, Los Angeles, CA 90095, USA

**Keywords:** fibrin, drug delivery, stimuli-responsive, factor XIII, enzymatic incorporation, bioconjugation, oligonucleotide conjugate, peptide conjugate, alpha 2-plasmin inhibitor

## Abstract

There are a limited number of stimuli-responsive biomaterials that are capable of delivering customizable dosages of a therapeutic at a specific location and time. This is especially true in tissue engineering and regenerative medicine applications, where it may be desirable for the stimuli-responsive biomaterial to also serve as a scaffolding material. Therefore, the purpose of this study was to engineer a traditionally non-stimuli responsive scaffold biomaterial to be thermally responsive so it could be used for on-demand drug delivery applications. Fibrin hydrogels are frequently used for tissue engineering and regenerative medicine applications, and they were functionalized with thermally labile oligonucleotide tethers using peptides from substrates for factor XIII (FXIII). The alpha 2-plasmin inhibitor peptide had the greatest incorporation efficiency out of the FXIII substrate peptides studied, and conjugates of the peptide and oligonucleotide tethers were successfully incorporated into fibrin hydrogels via enzymatic activity. Single-strand complement oligo with either a fluorophore model drug or platelet-derived growth factor-BB (PDGF-BB) could be released on demand via temperature increases. These results demonstrate a strategy that can be used to functionalize traditionally non-stimuli responsive biomaterials suitable for on-demand drug delivery systems (DDS).

## 1. Introduction

On-demand drug delivery is an important element of the precision medicine approach to treating disease or injury. Precision medicine accounts for biological variability between individuals and subsets of patients when treating diseases and injuries [1,2,3]. This includes differences in personal characteristics (e.g., age, weight, sex, etc.), environment, and genotype and phenotype information, which are known to affect drug sensitivity [4,5,6,7]. The advantages of on-demand drug delivery systems (DDS) in precision medicine are that they are capable of delivering customizable dosages at a specific location and time. Additionally, on-demand DDS can be used to deliver multiple drugs with complex release profiles. These drug cocktails are common in cancer treatment [8,9], immunosuppression [10], and regenerative pharmacology [11]. Several recent reviews of on-demand DDS examine the various strategies used for triggered release, which include leveraging electric and magnetic fields as well as electromagnetic radiation [12,13,14,15]. A significant limitation of these systems is the limited selection of stimuli-responsive biomaterials. This is especially true in tissue engineering and regenerative medicine applications where dynamic and responsive biomaterials can help recapitulate the native microenvironment of tissues [16]. This bottleneck limits the clinical impact of on-demand DDS in precision medicine, and adoption of on-demand drug delivery for this application requires expanding the library of biomaterials that are capable of responding to external stimuli and can be fabricated into different form factors (e.g., 3D scaffolds, films/membranes, micro- and nanospheres).

Fibrin is a popular scaffolding material for tissue engineering and regenerative medicine applications [17,18,19,20]; however, it is not stimuli-responsive. Previous studies have shown that fibrin and other naturally derived hydrogels as well as biomaterials composed of synthetically derived components can leverage biochemical reactions that happen in the coagulation cascade to independently modify the stiffness and/or present biologically active moieties [21,22,23,24,25,26,27]. This is accomplished by attaching substrate peptides for factor XIII (FXIII)—a transglutaminase that crosslinks glutamine and lysine residues within the hydrogel network—to the molecules of interest [28]. This study aimed to utilizing this strategy to functionalize fibrin with DNA oligonucleotide (oligo) tethers. Double-stranded DNA oligo de-hybridizes at elevated temperatures, and the temperature that de-hybridization occurs at can be modulated to physiologically relevant temperatures by changing the number of base pairings between the two strands as well as the guanine-cytosine content [29]. This strategy to use oligonucleotides as (photo)thermally responsive tethers for on-demand release has been previously reported [30,31,32,33]. For example, a previous study conjugated oligo tethers to gold nanoparticles, which generate heat upon exposure to near infrared light, in order to de-hybridize the double stranded oligo and trigger the release of single stranded oligo [34]. The advantages of light and heat as stimuli for on-demand DDS have been previously reviewed and includes high spatiotemporal resolution for light while heat is a unique stimulus since it can originate from either an internal (e.g., tumor) or external source [35,36]. By conjugating these tethers to fibrin, a stimuli-responsive delivery vehicle that is (photo)thermally controlled can be fabricated out of traditionally non-responsive biomaterials.

In this study, the incorporation efficiency of peptides from several FXIII substrates into fibrin hydrogels was investigated. FXIII has been found to have over 150 substrates in human plasma [37], but the list was limited to peptides from alpha 2-plasmin inhibitor, fibronectin, vitronectin, and von Willebrand factor for this study. Oligo tethers were evaluated for their ability to anchor and release on-demand both fluorophores (model small molecule drugs) as well as growth factors. Triggered release from the fibrin hydrogels was shown upon exposure to elevated temperatures.

## 2. Materials and Methods

### 2.1. Enzymatic Incorporation of Peptides into Fibrin Hydrogels

#### 2.1.1. Fabrication of Fibrin Hydrogels

The fibrinogen complex component of the Tisseel™ fibrin sealant kit (Baxter Healthcare Corp., BioScience, Westlake Village, CA, USA) was reconstituted with the aprotinin solution following the manufacturer’s instructions. Note this component is not a purified source of fibrinogen and may contain trace amounts of growth factors, enzymes, and extracellular matrix proteins [38]. Subsequent fibrinogen dilution was prepared by mixing the reconstituted fibrinogen complex with 1× Tris-buffered saline (TBS; MilliporeSigma, Burlington, MA, USA). The thrombin component of the Tisseel™ fibrin sealant kit was reconstituted and diluted with a solution of calcium chloride (3 mM or 40 mM) in TBS. At the time of experiment, 100 µL of fibrinogen solution was added to the hydrogel casting mold (i.e., cap of 1.5 mL microcentrifuge tube). Fibrin polymerization was initiated with the addition of 100 µL thrombin to the casting mold. Activated human FXIII (Innovative Research, Inc., Novi, MI, USA) was included in the thrombin solution so that each fibrin hydrogel contained 1 µg of FXIII. The fibrin solutions were gently mixed to produce a uniform fibrin construct and were incubated at 37 °C for 30 min to allow the formation and stabilization of the hydrogels. The fibrin hydrogels were soaked in TBS for 24 h to remove any unreacted constituents.

#### 2.1.2. Comparing FXIII Substrate Peptide Incorporation

The incorporation efficiency of the following FXIII substrate peptides modified with 5-carboxyfluorescein: NQEQVSPL (alpha 2-plasmin inhibitor; CPC Scientific, Sunnyvale, CA, USA) [21], RQAQQMVQPQ (fibronectin; CPC Scientific) [39], NPEQTPVL (vitronectin; CPC Scientific) [40], and TCQSLHIN (von Willebrand factor; CPC Scientific) [41], into fibrin hydrogels during enzymatic polymerization was determined by measuring the unincorporated fluorescently tagged peptides that leached from the fibrin hydrogel during the 24 h soak in TBS and subtracting it from the peptide amount initially added to the gel then dividing by the initial amount. Fibrin hydrogels were prepared as described above with the following modifications: FXIII substrate peptide was added to the fibrinogen complex solution so there was 1 nmol of peptide added to each hydrogel. The final fibrin concentration was 2.5 mg/mL (0.68 nmol peptide/1 nmol fibrin) with 2 IU/mL of thrombin. The polymerization reaction occurred at pH 7.4. The fluorescence was measured using a spectrophotometer (Ex: 485 nm/Em: 535 nm; Tecan Infinite F200 plate reader, Mannedorf, Switzerland).

#### 2.1.3. Polymerization Reaction Conditions’ Effect on Peptide Incorporation

The effect that reaction conditions and constituent concentrations had on peptide incorporation efficiency and the resulting hydrogel network was determined by measuring the unincorporated fluorescently tagged peptides (NQEQVSPL modified with 5-carboxyfluorescein), as previously described, and the mass swelling ratio, respectively. Fibrin hydrogels were prepared as described above with the following modifications: reconstitution and dilutions were made with 1× TBS at pH 6.5, 7.4, or 8.4; the final fibrin concentration was either 2.5 mg/mL or 5 mg/mL; and the mole of peptide to mole of fibrinogen ratio was either 0.34, 0.68, 3.40, or 6.80. To calculate the mass swelling ratio, the polymerized fibrin hydrogels were allowed to freely swell in di-H_2_O for 24 h and the swollen hydrogel mass was then measured. Hydrogels were then flash-frozen in liquid N_2_ and lyophilized, and then the dry mass was measured. The mass swelling ratio is reported as the ratio of swollen mass to dry mass.

### 2.2. Thermally Triggered Release of Complement Oligonucleotide

To demonstrate thermally triggered release, oligonucleotide tethers comprised of a parent strand and a complement strand were conjugated to the surface of a 96-well plate and then exposed to elevated temperatures to trigger release.

#### 2.2.1. Annealing Oligonucleotides

Modified ‘parent’ oligo strands with a S-trityl-6-mercaptohexyl derivative protecting group and a 6-carbon spacer arm to separate the oligo from the thiol group (5′-Thiol Modifier (C6 SS)—GAA GTG CGG TTA GTC GGC *TTG AAT CAG CGA*-3′; MilliporeSigma) were deprotected in a 100mM dithiothreitol (DTT) solution (pH 8; MilliporeSigma) for 3 h at room temperature and purified using NAP-10 size-exclusion columns (Cytiva, Marlborough, MA, USA) equilibrated with PBS (pH 7.4). ‘Complement’ oligo strands (5′-TAT AAG *TCG CTG ATT CAA*-3′; MilliporeSigma) with 12 overlapping base pairs (italicized) were added to the parent oligo solution (1:1 molar ratio), heated to 70 °C for 10 min, cooled to room temperature, and allowed to anneal overnight.

#### 2.2.2. Functionalization of Substrate

The wells were coated with chitosan (MilliporeSigma) dissolved in acetic acid (1N; ThermoFisher Scientific, Somerset, NJ, USA) by adding 200 μL of a 2% *w/v* chitosan solution to the wells and leaving the plate overnight in the fume hood to dry. The plates were subsequently placed in a desiccator until completely dried. Prior to conjugation, the surface was washed 3× with 200 μL of sodium hydroxide (1N; MilliporeSigma) and followed by 3× washes with 1× phosphate buffer solution (PBS; ThermoFisher Scientific).

Annealed oligo was conjugated to the chitosan-coated surface using sulfosuccinimidyl 4-(N-maleimidomethyl)cyclohexane-1-carboxylate (sulfo-SMCC, MilliporeSigma) in a two-step reaction. The chitosan-coated surface was reacted with the crosslinker for 2 h at 25 °C [42,43] and rinsed 3× with 1× PBS to remove excess crosslinker; then, annealed oligo was added and reacted overnight at 25 °C. The surface was rinsed 3× with 1× PBS to remove unreacted oligo.

#### 2.2.3. Triggered Release of Fluorescently Labeled Complement Oligonucleotide

Triggered release of complement oligo labeled with Texas Red from the substrate into the supernatant (1× PBS) was performed by heating the plate to 45 °C for 20 min at the 80 min and 160 min time points. After heating, the plate was allowed to cool to room temperature. At 20 min intervals over 4 h, the supernatant was collected and replaced with 1× PBS, and the fluorescent intensity of both the functionalized substrate and supernatant were measured using a spectrophotometer (Ex: 535 nm/Em: 565 nm; Tecan Infinite F200 plate reader). Texas Red conjugation was performed by the oligo supplier and occurred at the 5′ end.

#### 2.2.4. Triggered Release of Growth Factor-Oligonucleotide Conjugate 

Platelet-derived growth factor-BB (PDGF-BB, MilliporeSigma) was first reacted with 1 mg/mL sulfo-SMCC linker for 2 h. The PDGF-BB-linker solution was then reacted with I-125 (MP Biomedicals, Irvine, CA, USA) in a 50 mM HEPES buffer (MilliporeSigma) for 15 min. The sample was then isolated with column chromatography. Elutions were checked for radioactivity with a Cobra Auto-Gamma Counter (Model B5002, Packard Instrument Company, Meriden, CT, USA).

Radio-labeled PDGF-BB was first conjugated to complement oligo with a 5′ S-trityl-6-mercaptohexyl derivative-protecting group and a 6-carbon spacer arm to separate the oligo from the thiol group that was deprotected using DTT as previously described. The thiol on the complement oligo was reacted overnight in 50 mM HEPES at 25 °C with maleimide groups introduced on PDGF-BB via sulfo-SMCC. The PDGF-complement oligo conjugate was then annealed with the parent oligo (1:1 molar ratio) overnight by heating the solution to 70 °C for 10 min and allowing it to cool to room temperature. The PDGF-annealed oligo complex was then conjugated to a chitosan-coated surface using the same two-step reaction with sulfo-SMCC as previously described. Unconjugated PDGF-BB was removed by an overnight wash using 1× PBS.

Triggered release of complement oligo with radio-labeled PDGF-BB from the substrate into the supernatant (1× PBS) was performed by heating to 45 °C every 24 h. The supernatant was measured for radioactive activity immediately before and after heating with a Cobra AutoGamma Counter. This was repeated until the surface had been exposed to 5 heating cycles. The first two heating cycles were 5 min long, and the remaining cycles were 10 min long.

### 2.3. Thermally Triggered Release of Complement Oligonucleotide

#### 2.3.1. Oligonucleotide-Peptide Conjugate Synthesis 

**Figure 1:**
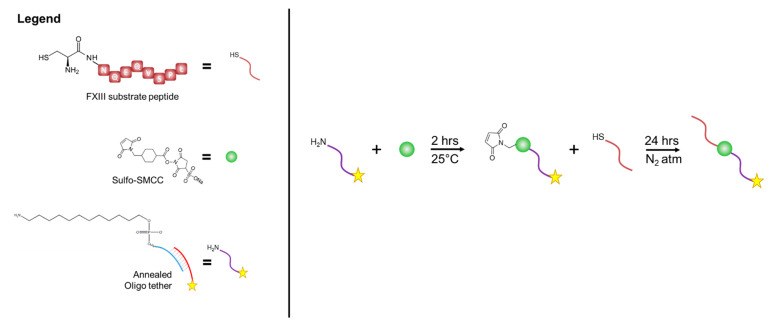
Oligonucleotide–peptide conjugate reaction scheme. The amine-modified parent oligo is first reacted with the N-hydroxysuccinimide ester on the heterobifunctional crosslinker: sulfo-SMCC. The FXIII substrate peptide containing a thiol group via the addition of cysteine residues was then reacted with the maleimide group on the sulfo-SMCC crosslinkers attached to the annealed oligo.

Oligo-peptide conjugates were synthesized through several steps (Figure 1). First, the parent and complement oligo strands were annealed by combining in a HEPES buffer at 1:1 molar ratio and heating to 100 °C for 1 h, followed by cooling to room temperature. For this experiment, the 5′ end of the ‘parent’ oligo strands were modified with a C12-amine modifier, which at the time of the experiment was reacted with sulfo-SMCC crosslinker. Ethanol precipitation was used to remove unreacted crosslinker, and the annealed oligo were resuspended in TBS (pH = 7). FXIII substrate peptides containing thiol groups via the addition of cysteine residues (CNQEQVSPL; CPC Scientific) were used to react with the maleimide group on the sulfo-SMCC crosslinkers attached to the annealed oligo. Any disulfide bridge between cysteine residues on the peptides was reduced using DTT, and excess DTT was removed using a NAP-10 column with a monopotassium phosphate mobile phase. The reduced peptide solution was then added to the oligo-maleimide solution at a 1:1 mole ratio and incubated for 24 h under an inert atmosphere.

#### 2.3.2. Enzymatic Incorporation of Oligonucleotide-Peptide Conjugate into Fibrin Hydrogels

To avoid physical entrapment of unreacted oligo-peptide conjugate in the fibrin hydrogel network, pre-fabricated fibrin hydrogels—prepared as previously described—were soaked in a FXIII solution containing 1 µg of the enzyme with fluorescein labeled oligo-peptide conjugates for 24 h at room temperature to enzymatically incorporate the conjugate into the hydrogels. Prior to and following incubation with fibrin hydrogels, the fluorescent intensity of the enzyme-conjugate solution was measured using a spectrophotometer to quantify initial (C_o_) and final conjugate concentration (C_f_), respectfully. Following enzymatic incorporation, the fibrin hydrogels were soaked in fresh 1× TBS for 24 h at room temperature to remove any conjugate that had non-specifically adsorbed to the hydrogels. The fluorescent intensity of the supernatant was measured to quantify the non-specifically adsorbed conjugate (C_n_). The enzymatic incorporation of the conjugate into the fibrin hydrogels was calculated using Equation (1).

[(C_o_ − C_f_ − C_n_)/C_o_] × 100% (1)

#### 2.3.3. Thermally Triggered Release of Complement Oligonucleotide Strands from Fibrin Hydrogels

Release studies were conducted in 1× TBS (pH = 7). Each fibrin hydrogel was heated to 50 °C for 10 min intervals. The supernatant was subsequently collected, and the fluorescent intensity was measured using a spectrophotometer. To assess whether released complement strands would re-anneal with anchored parent oligonucleotide strands, the supernatant containing released complement strands was returned to the fibrin hydrogels and allowed to cool to room temperature. The fibrin hydrogels were then exposed to an additional 10 min heating cycle (50 °C), and the amount of fluorescently tagged complement oligonucleotide strands re-released during the heating cycle was measured using a spectrophotometer.

### 2.4. Statistical Analysis

Statistical analysis was performed with GraphPad Prism 9.3.0 software. A two-way analysis of variance (ANOVA) with Tukey’s post hoc test was used to analyze the difference between the means of more than two groups. A Student’s *t*-test was used to compare the means between two groups. *p*-values of less than 0.05 were considered statistically significant. All values are reported as mean ± standard deviation.

## 3. Results

### 3.1. FXIII Substrate Peptide Incorporation into Fibrin Hydrogels

Comparison of the incorporation efficiency for various FXIII substrate peptides showed that the peptide from alpha 2-plasmin inhibitor had the greatest incorporation efficiency (Table 1). The peptide from von Willebrand factor had the second most incorporation into the fibrin hydrogels; however, there was significant non-specific adsorption of the peptide since there was no significant difference in incorporation efficiency when compared to negative control hydrogels that were prepared without exogenous FXIII added to the reaction mixture (*p* = 0.9). Similarly, there was no significant difference in fibronectin and vitronectin peptide incorporation compared with their respective negative control hydrogels. Peptide incorporation increased with increasing calcium concentration in the reaction mixture; however, only for the alpha 2-plasmin inhibitor peptide was the increase from 1.5 to 20 mM calcium significant (26.3% ± 1.1% vs. 36.9% ± 1.8%; *p* < 0.0001).

**Figure 2:**
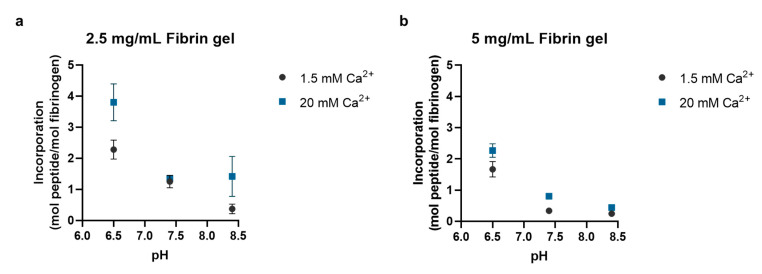
The pH-dependent enzymatic incorporation efficiency of the alpha 2-plasmin inhibitor peptide into fibrin hydrogels prepared with (**a**) 2.5 mg/mL fibrinogen and (**b**) 5 mg/mL fibrinogen. Increasing the pH of the fibrin polymerization reaction decreased the peptide incorporation while increasing the Ca^2+^ concentration in the reaction increased the peptide incorporation. For all conditions, the fibrin hydrogels were prepared with 2 IU/mL thrombin and incubated at 37 °C for 30 min to allow the fibrin hydrogels to polymerize (*n* = 3).

The effect pH had on the enzymatic incorporation of the alpha 2-plasmin inhibitor peptide into fibrin hydrogels with different fibrin concentrations is shown in Figure 2. Overall, the peptide incorporation decreased with increasing pH. In the 2.5 mg/mL fibrin hydrogels (Figure 2a), peptide incorporation was significantly greater when the reaction was run at pH 6.5 than the incorporation achieved by running the reaction at pH 8.4 in the presence of 1.5 mM Ca^2+^ (2.3 ± 0.3 vs. 0.4 ± 0.3 mol peptide/mol fibrinogen; *p* = 0.0007). A similar result was observed in the 5 mg/mL fibrin hydrogels prepared with 1.5 mM Ca^2+^ (Figure 2b). Peptide incorporation was significantly greater when the pH was acidic during hydrogel fabrication versus basic (1.7 ± 0.2 vs. 0.2 ± 0.06 mol peptide/mol fibrinogen; *p* < 0.0001). Increasing the Ca^2+^ concentration again increased the peptide incorporation for both fibrin hydrogels. In the 2.5 mg/mL fibrin hydrogels, there was a significant increase in peptide incorporation under acidic conditions when the Ca^2+^ concentration increased from 1.5 to 20 mM (2.3 ± 0.3 vs. 3.8 ± 0.6; *p* = 0.005), and the increase in peptide incorporation under acidic conditions with increasing Ca^2+^ concentration was also significant in the 5 mg/mL fibrin hydrogels (1.7 ± 0.2 vs. 2.3 ± 0.2; *p* = 0.002).

**Figure 3:**
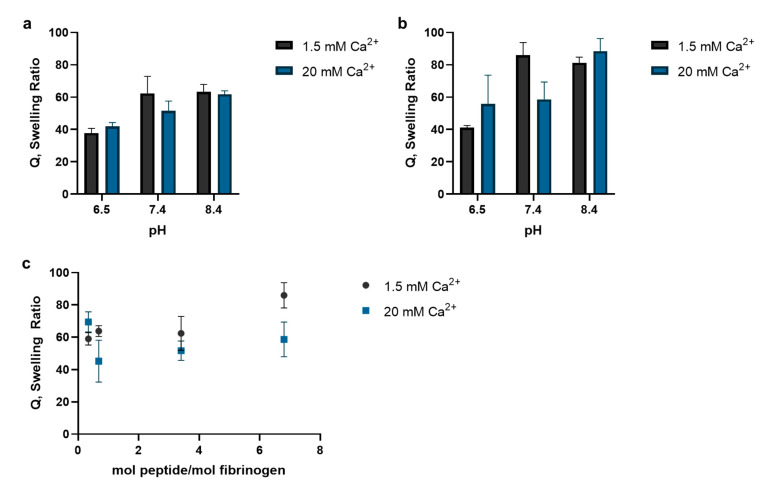
The pH-dependent mass swelling ratio (Q) of fibrin hydrogels following alpha 2-plasmin inhibitor peptide incorporation into fibrin hydrogels prepared with (**a**) 3.4 and (**b**) 6.8 mole ratio between alpha 2-plasmin inhibitor peptide and fibrinogen. Increasing the pH of the fibrin polymerization reaction increased Q, while there was a decreasing trend in Q with increasing Ca^2+^ concentration in the reaction. (**c**) Holding the pH constant at 7.4 and increasing the ratio between alpha 2-plasmin inhibitor peptide and fibrinogen increased the mass swelling ratio. For all conditions, the fibrin hydrogels were prepared with 2 IU/mL thrombin and incubated at 37 °C for 30 min to allow the fibrin hydrogels to polymerize (*n* = 3).

Analysis of the swelling ratio revealed that preparing hydrogels in neutral to basic reaction environment increased the mass swelling ratio of the resulting hydrogel, as did having a greater the mole ratio between alpha 2-plasmin inhibitor peptide and fibrinogen at the start of the reaction (Figure 3). In the hydrogels with a 3.4 mole ratio between alpha 2-plasmin inhibitor peptide and fibrinogen, there was no statistically significant difference in the mass swelling ratios between any of the hydrogels prepared in the basic (pH = 8.4) and neutral (pH = 7.4) conditions (Figure 3a). However, there was a statistically significant difference in the mass swelling ratio between the hydrogels prepared in the basic (pH = 8.4) versus acidic (pH = 6.5) conditions (*p* < 0.01). A similar trend was observed when the mole ratio between alpha 2-plasmin inhibitor peptide and fibrinogen was increased to 6.8 (Figure 3b). For example, the mass swelling ratio of the fibrin hydrogels prepared with 1.5 mM Ca^2+^ in the acidic condition (Q = 41.1 ±1.3) was significantly less than the hydrogels prepared in the neutral (Q = 85.9 ±7.9, *p* = 0.001) and basic (Q = 81.3 ±3.4, *p* = 0.003) conditions. Holding the pH constant at 7.4 and increasing the ratio between alpha 2-plasmin inhibitor peptide and fibrinogen increased the mass swelling ratio (Figure 3c). Hydrogels prepared with 1.5 mM Ca^2+^ had greater mass swelling ratios than the fibrin hydrogels prepared with 20 mM Ca^2+^ with the exception of the 0.34 mole ratio condition, but the difference was not statistically significant.

### 3.2. Thermally Triggered Release of Complement Oligonucleotide

**Figure 4:**
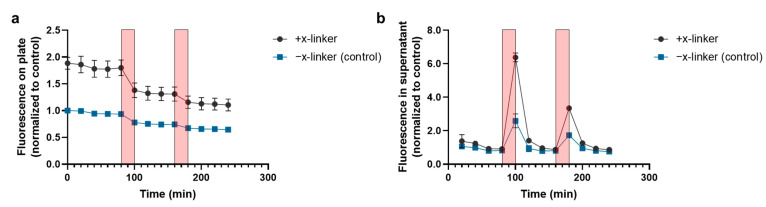
Time-dependent fluorescent intensity of (**a**) the chitosan-coated substrate and (**b**) the supernatant, which were exposed to two heating cycles (red boxes). The fluorescent intensity was normalized to the control group (−x-linker; non-specifically adsorbed oligonucleotide) (*n* = 3).

Thermally triggered release of fluorescently tagged complement oligo from the parent oligo, which was covalently bonded to a chitosan-coated substrate, is shown in Figure 4. The fluorescent signal on both the chitosan-coated substrate (Figure 4a) as well as in the supernatant (Figure 4b) were measured. Heating the chitosan-coated substrates functionalized with annealed oligo tethers produced a significant release of fluorescence signal into the supernatant when compared to the release from the negative control substrate—annealed oligo tethers non-specifically adsorbed to the chitosan-coated substrate (heat cycle 1: 6.4 ± 0.3 vs. 2.6 ± 0.4-fold increase over baseline release; *p* < 0.0001; heat cycle 2: 3.3 ± 0.06 vs. 1.7 ± 0.1-fold increase over baseline release; *p* < 0.0001). There was a significant drop in the fluorescent signal from the conjugated surface that coincided with the spike in fluorescent signal in the supernatant from the first heat cycle (*p* < 0.0001), but not for the second heat cycle (*p* = 0.9). For the negative control substrate, there was no significant difference in the fluorescent signal before and after either heating cycle. Following the heating cycles, the release of fluorescently tagged complement oligo into the supernatant returned to baseline levels as the substrate cooled to room temperature.

**Figure 5:**
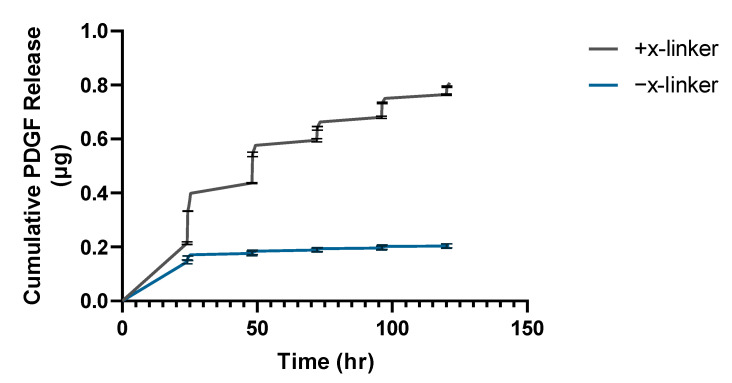
The cumulative release (μg) of PDGF-BB (24.3 kDa) from chitosan-coated substrates triggered by heat exposure. Heating cycles occurred every 24 h, starting at t = 24 h. After 5 heating cycles, nearly 60% of the PDGF-BB remained on the substrate. The negative control group (−x-linker) measured the growth factor–oligo conjugate that had non-specifically adsorbed to the substrate, and nearly all of it was collected during the first 24 h (*n* = 3).

The triggered release profile of radio-labeled PDGF-complement oligo conjugate released from chitosan-coated substrates is shown in Figure 5. To account for non-specific adsorption of the growth factor-oligo conjugate to the substrate, chitosan-coated substrates that did not have the growth factor-tether conjugate covalently bonded via the sulfo-SMCC crosslinker served as the negative control. Both substrates showed an initial burst release within the first 24 h: 0.15 μg (±0.005 μg) from the control substrate, and 0.22 μg (±0.008 μg) from the bonded substrate. The first heat exposure (5 min at 45 °C) triggered the release of 0.12 μg of PDGF-complement oligo from the bonded substrate compared to 0.014 μg from the control substrate. The second, third, fourth, and fifth exposures triggered the release of 0.11, 0.045, 0.054, and 0.029 μg of PDGF-complement oligo conjugate from the bonded substrate, and 0.004, 0.002, 0.003, and 0.001 μg from the control substrate, respectively. In total, 0.81 μg (±0.001 μg) of conjugate was released from the substrate over 5 days with thermal exposure every 24 h, which was significantly greater than the 0.21 μg (±0.008 μg) released from the control substrate (*p* < 0.0001). At the start of the triggered release study, there was 1.98 ± 0.25 μg of PDGF-BB tethered to the chitosan-coated substrate, and after the five heating cycles over 120 h, nearly 60% of the radio-labeled PDGF-BB remained on the substrate. There was initially 0.25 ± 0.08 μg of PDGF-BB non-specifically absorbed to the negative control substrate, and nearly all of it was collected during the 5 heating cycles over 120 h.

### 3.3. Thermally Triggered Release from Fibrin Hydrogels

**Figure 6:**
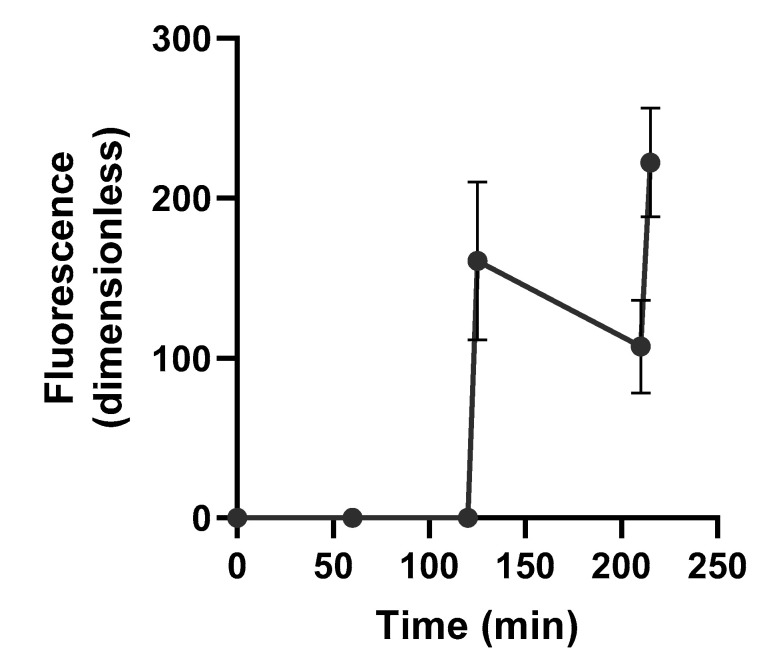
The time-dependent fluorescent intensity of supernatant from immersed fibrin hydrogels exposed to two 5 min heating cycles at t = 120 and 210 min. The supernatant was returned to the immersion solution following measurement and allowed to cool to room temperature prior to a second heating cycle (*n* = 3).

The functionalized fibrin hydrogels demonstrated thermally triggered release when exposed to elevated temperatures (50 °C) for 5 min (Figure 6). Prior to heat exposure, there were no detectable levels of fluorescence from the labeled complement oligo detected in the supernatant for the first 2 h. Exposure to heat produced a spike in the fluorescence of the supernatant, indicating the presence of complement oligo in the supernatant. As the hydrogels and supernatant cooled to room temperature, there was a 33.3% decrease in the supernatant’s fluorescence (*p* = 0.05). After the second exposure, there was a 107% increase in the supernatant’s fluorescence (*p* = 0.004). The fluorescence signal following the second heat exposure was greater than the reading following the first heat exposure; however, there was no statistically significant difference between the fluorescent readings at t = 125 and 215 min, which immediately followed the heat exposures (*p* = 0.06).

## 4. Discussion

The aim of this study was to functionalize fibrin hydrogels with thermally responsive oligo tethers. To do this, various glutamine-containing peptides from known FXIII substrates were evaluated for their enzymatic incorporation efficiency. These substrate peptides included segments from alpha 2-plasmin inhibitor [21], fibronectin [39], vitronectin [40], and von Willebrand factor [41]. Alpha 2-plasmin inhibitor peptide showed the greatest incorporation efficiency out of the peptides studied, while little-to-no incorporation of the other candidate substrate peptides was detected, despite having known crosslinking sites for FXIII. Differences in the crosslinking reaction rates as well as the presence or absence of key amino acids in the peptide may explain the difference. For instance, a previous study found that the rate of alpha 2-plasmin inhibitor crosslinking to fibrin during the first 6 min was nearly twelvefold greater when asparagine was at the N-terminal versus methionine [44]. However, additions, subtractions, and substitutions of amino acids to the substrate peptides and the effect on incorporation efficiency were not included in this study. Consequently, there may be amino acids missing from the group of peptides studied here that may have significantly impacted their crosslinking to the fibrin hydrogels. Because of the important role alpha 2-plasmin inhibitor plays in protecting the fibrin clot from fibrinolysis, the successful incorporation of this peptide is reasonable, and it was selected for further investigation.

To maximize peptide incorporation efficiency, the impact reaction conditions had on incorporation of the alpha 2-plasmin inhibitor peptide was studied. The results showed that there was decreased incorporation efficiency with increasing pH. The active site of FXIII includes a cysteine residue that initiates the crosslinking reactions by forming a thioester bond with glutamine [45,46]. However, at pH 8.4, the thiol group on cysteine’s side chain is deprotonated (pK_a_ = 8.3) and prone to forming disulfide bridges with other thiol groups, which would inactivate the enzyme. The hydrogels formed under basic conditions had the greatest mass swelling ratios, which could be an indicator of decreased crosslinking between chains in a hydrogel network [47]. These results support the hypothesis that decreased peptide incorporation at pH 8.4 is due to inactivation of FXIII, since inactive FXIII would also not be able to crosslink neighboring fibrin chains. Calcium concentration was another important variable impacting peptide incorporation, with increased calcium concentrations being associated with increased peptide incorporation. Calcium binding to sites on FXIII facilitate conformational changes that form substrate-binding pockets and ultimately expose the active site [48]. The reaction conditions that maximized peptide incorporation were subsequently used for the reactions that functionalized fibrin with the oligo tethers.

There are other variables that were not included in this study but could impact peptide incorporation efficiency. For instance, the thrombin concentration was held constant during these experiments. In addition to cleaving the fibrinopeptides that subsequently allow fibrinogen to polymerize and form fibrin, thrombin is responsible for activating FXIII. In this study, however, pre-activated FXIII was used. The amount of FXIII added to the hydrogels was also kept constant. Furthermore, the full practical range of concentrations for the variables included in these experiments were not studied. All these variables create an extremely large test space that is laborious and resource-intensive to analyze using the one-factor-at-a-time approach. Statistical approaches, such as response surface methodology, simplify the screening process by utilizing statistical analysis of collected data to identify the variables that significantly affect the incorporation efficiency and calculate a randomized list of formulations aimed at optimizing that outcome. After performing screening experiments, the classical approach could be used to isolate the optimized construct formulation.

Triggered release of complement oligo from parent oligo that was conjugated to an aminated substrate was used to demonstrate that double stranded oligonucleotides were suitable thermally responsive tethers for growth factor release. Previous studies have demonstrated thermally triggered release using oligonucleotide tethers, but only with fluorophores as model drugs [30,31,34]. Exposing surface-immobilized oligo tethers to heat significantly increased the levels of radiolabeled PDGF-BB in the supernatant while little to no release occurred in the absence of heat, suggesting that dehybridization of the compliment oligo from the parent oligo at elevated temperatures was responsible for the growth factor release. These results confirmed that oligo tethers are capable of releasing a therapeutically significant protein. Growth factor function while attached to the complement oligo was not studied here, and it is not known whether removal of the oligo tether post-triggered release is required to preserve growth factor function.

Fluorescently tagged oligo-peptide conjugates were successfully incorporated into fibrin hydrogels enzymatically. By measuring the unincorporated conjugate remaining in the supernatant, it was determined that nearly 40% of the conjugate was attached to the fibrin hydrogels by 1 μg of FXIII. The conjugates were enzymatically attached to pre-fabricated fibrin to avoid physical entrapment of unreacted oligo-peptide conjugate in the hydrogel network that would slowly release from the hydrogel via diffusion. As such, there was no oligo released from the fibrin hydrogels prior to heat exposure. Triggered release of fluorescein-tagged complement oligo was measured in the supernatant following exposure to elevated temperatures. These findings strongly suggest that de-hybridization of oligo tethers caused by the increased thermal energy and release of the complement oligo was responsible for the fluorescent spike measured in the supernatant. Furthermore, as the hydrogels and supernatant cooled back to room temperature, there was a decrease in the supernatant’s fluorescence, which suggests some of the released complement oligo strands re-annealed with parent oligo strands that are covalently attached to the hydrogel network. Subsequent heat exposure produced another spike in released complement oligo that was greater than the first spike. Because oligo de-hybridization is reversible, repeat triggered release is possible from these functionalized fibrin hydrogels. Additionally, melting of double stranded oligo occurs over a narrow temperature range rather than an all-or-nothing event. Therefore, residual oligo tethers that have not de-hybridized are expected to be present in the hydrogel network and available for subsequent triggering events.

The temperatures used to trigger release in this study (i.e., >45 °C) have the potential to damage tissue and negatively impact growth factor folding. It is possible that because an external heating source was used to heat the hydrogels rather than an internal heat source (e.g., the photothermal response of entrapped chromophores exposed to irradiation), higher temperatures were required to trigger release. Additionally, there are several design strategies that can be employed to change the melting temperature and curve of the oligo tethers. A previous study found that the placement of guanine–cytosine (G/C) base pairs in an otherwise adenine–thymine (A/T) strand impacted both the melting temperature and the melting curve [49]. Specifically, the strands with G/C ends had a lower melting temperature and a sharp sigmoid melting curve compared to the G/C core and G/C mix strands, which had melting temperatures nearly 10 °C greater and shallower sigmoid melting curves. Furthermore, it has been shown that the oligo tether length as well as the use of a supernatant that more closely mimics the in vivo environment (i.e., presence of ions) all impact the melting temperature [31,34,50]. The impact these three factors have on the salt-adjusted melting temperature were calculated with the Oligonucleotide Properties Calculator created by Northwestern University (data not shown) [51]. Given the Na^+^ concentration in human blood is approximately 140 mM, it is expected that the melting temperature with the oligonucleotide sequence used in this study would be greater than the target 45 °C. Furthermore, changes in melting temperature vary more with oligo length and G/C content. Clearly, the de-hybridization temperature of the oligo tethers can be tailored to the target physiological temperature range by changing the number of base pairs as well as the guanine–cytosine content.

The strategy employed here to outfit fibrin hydrogels with thermally responsive tethers is amendable to other biopolymers used in tissue engineering and regenerative medicine applications—each having unique advantages and disadvantages. For instance, alginate is biocompatible and demonstrates long term stability in vivo [52], but lacks cell binding sites and would require modification with RGD to promote cell survival if the delivery vehicle is intended to serve as a scaffold as well [53]. Additionally, each biomaterial will have unique properties that could be exploited for the incorporation of thermally responsive tethers, and there are several classes of therapeutics that could be delivered on-demand (e.g., small molecules, nucleic acids, peptides, and proteins). Identifying novel ways to engineer traditionally non-stimuli responsive biomaterials to be used in on-demand DDS would help to advance the adoption of precision medicine in a broad range of healthcare disciplines.

## 5. Conclusions

This study demonstrates that double-stranded oligonucleotide tethers can be enzymatically incorporated into fibrin hydrogels and the single-strand complement oligo can be released on-demand by a temperature change. The alpha 2-plasmin inhibitor peptide had the greatest incorporation efficiency out of the peptides studied at 37% when 20 mM Ca^2+^ was in the reaction solution and could be conjugated to the parent oligo strand using the heterobifunctional crosslinker sulfo-SMCC. Nearly 40% of this conjugate was successfully incorporated into fibrin hydrogels via the activity of FXIII. Furthermore, the data demonstrate that oligonucleotide tethers are capable of tethering and releasing PDGF-BB on demand. Future studies investigating whether growth factors retain their bioactivity following conjugation to the complement oligo and triggered release are necessary to support the applicability of stimuli-responsive hydrogels for tissue engineering and regenerative medicine applications. Ultimately, the results provide a strategy to expand the library of stimuli responsive biomaterials for on-demand drug delivery.

## Figures and Tables

**Table 1:** Incorporation of FXIII substrate peptides into 2.5 mg/mL fibrin hydrogels.

Peptide ^3^	CaCl_2_ Conc. (mM)	Incorporation (%) ^1^	
		+FXIII ^2^	−FXIII
NQEQ	1.5	26.3% (±1.1%)	0.3% (±0%)
	20	36.9% (±1.8%)	0.4% (±0%)
RQAQQ	1.5	0.5% (±0.1%)	0.1% (±0%)
	20	0.8% (±0.1%)	0.1% (±0%)
NPEQ	1.5	1.2% (±0%)	0.1% (±0%)
	20	1.6% (±0.2%)	0.1% (±0%)
TCQS	1.5	4.0% (±0.1%)	3.5% (±0.1%)
	20	4.6% (±0.1%)	4.3% (±0.1%)

^1^ Reaction conditions: pH = 7.4; temp = 37 °C (n = 3). ^2^ 1 µg of FXIII added to the reaction mixture. ^3^ 1 nmol of peptide added to the reaction mixture.

## Data Availability

The data presented in this study are available on request from the corresponding authors.
